# Improved Positive Predictive Performance of *Listeria* Indicator Broth: A Sensitive Environmental Screening Test to Identify Presumptively Positive Swab Samples

**DOI:** 10.3390/microorganisms7050151

**Published:** 2019-05-27

**Authors:** Alan D. Olstein, Joellen M. Feirtag

**Affiliations:** 1 Paradigm Diagnostics, Inc., 800 Transfer Road Suite 12, St. Paul, MN 55114, USA; 2 Department of Food Science and Nutrition, University of Minnesota, St. Paul, MN 55108, USA; jfeirtag@umn.edu

**Keywords:** food safety, environmental *Listeria*, *Listeria* detection

## Abstract

PDX-LIB, *Listeria* Indicator Broth, was developed as a proprietary sensitive screening test to identify presumptively positive environmental swab samples for *Listeria* sp. The original formulation, while sensitive, initially proved to exhibit acceptable levels of false positive test results. Paradigm Diagnostics has been undertaken to modify the medium formulation to render it more selective while not sacrificing its sensitivity. After identification of a candidate formulation through laboratory studies, a field trial was conducted to validate the test performance parameters, including the true positive frequency and false positive frequency in several different food-processing facilities. Identical swab samples were enriched in both the original medium formulation and the new formulation. Presumptive positive samples were confirmed by plating on selective differential agar and qPCR analysis. The field trial data demonstrate that the new formulation significantly reduces the frequency of false positive samples compared to the original *Listeria* Indicator Broth formulation, without compromising the sensitivity of the original formulation. The new medium formulation resulted in no false positive samples compared to the 54% increased presumptive positive samples obtained with the original medium formulation.

## 1. Introduction

In a risk assessment study, the U.S. Department of Agriculture Food Safety Inspection Service provided the rationale for mandating a national surveillance program for *Listeria* occurrence in USDA-regulated facilities [1]. These new regulations mandated environmental surveillance for the presence of *Listeria* sp. in food processing facilities to minimize the risk of foodborne illness associated with contaminated food. This development impelled many firms, including Paradigm Diagnostics, to develop simple *Listeria* screening tests to enable the growing demand for this test volume [2].

**Figure 1:**
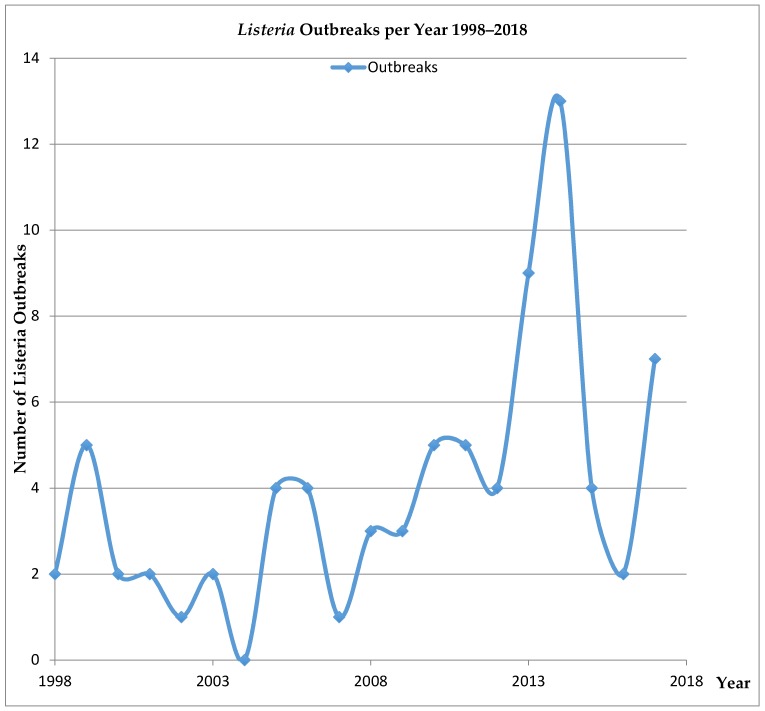
Listeria Outbreaks in the U.S. 1998–2018*. * From the NORS Dashboard Available at https://wwwn.cdc.gov/norsdashboard/. (Accessed on 9 May 2019).

A comprehensive study by the Center for Disease Control in 2012 provided evidence that the implementation of environmental controls in food processing facilities coupled with robust public health monitoring (Pulse Net) helped to reduce the burden of foodborne Listeriosis [3]. Despite these encouraging results, foodborne illnesses due to pathogens, including *Salmonella*, STEC, and *Listeria*, continue to be a challenge in the national food production system [4,5,6]. Figure 1 demonstrates that the frequency of Listeriosis outbreaks in the US has experienced a marked increase in the past few years. Consequently, accurate simple screening methods for foodborne illness pathogens must be available to address the on-going need for facility environmental surveillance.

In this study, we intend to demonstrate that an improved *Listeria* enrichment formulation can help to eliminate uncertainty when screening environmental samples for the presumptive presence of *Listeria* sp. Field trial data collected from eight different food-processing facilities supports the laboratory data, showing that the new formulation, LIB v.2.0, is more accurate than the antecedent test, LIB. Specifically, the false positives observed using LIB were completely eliminated using LIB v.2.0 without a loss of sensitivity for the detection of true *Listeria* positive samples. Appendix A was included to provide detailed location information of where the samples were obtained.

## 2. Materials and Methods

PDX-LIB and *Listeria* Indicator Broth v.2.0 and Securswabs were supplied by Paradigm Diagnostics, Inc. St. Paul, MN. Swabs were collected as duplicates from the same locations in food processing facilities and enriched in 20 mL of either LIB, the original formulation, or LIB v.2.0, the new medium formulation, for 48 h at 37 °C. Blackened samples were streaked onto modified MOX (modified Oxford) medium and incubated for an additional 18 h at 37 °C. The modified MOX medium was prepared by substituting the esculin in the standard MOX formulation with 5 g/L D-arabitol and 0.02 g/L bromcresol purple as the indicator system for *Listeria* sp. [7].

MOX-positive plates were confirmed as *Listeria* sp. by qPCR using primers and probes as detailed in the Food and Drug Administration Bacteriological Assay Manual [8]. Statistical analysis was conducted and pairwise comparisons between pathogen isolation rates using LIB v2.0 and LIB (original formulation) were made using the Mantel-Haenszel chi-square formula for unmatched test portions [9]. A Chi-Square value of less than 3.84 was considered to indicate no significant numerical difference between the two methods being compared. The formula for χ^2^ is

χ^2^= (|a-b|-1)^2^/(a+b)

a = The number of presumptively positive samples using LIB v.2.0.
b = The number of presumptively positive samples using LIB.

## 3. Results

A total of 161 samples were obtained from eight different food-processing facilities. Presumptive positive samples were identified and confirmed. Table 1 summarizes the results of field trial samples. Of the 161 environmental samples, LIB v-2.0 yielded 35 presumptive positives, while the original formulation resulted in 55 blackened samples. The 35 LIB v-2.0 samples were confirmed as true positives by plating and PCR analysis.

The LIB (original formulation) results yielded 54 presumptive positives, of which 35 were confirmed. Twenty of the presumptive positive LIB samples were deemed false positives. One hundred and seven of the LIB samples were negative, of which 106 were true negatives. One of the negative LIB samples was deemed a false negative since the duplicate LIB v-2.0 sample yielded a true positive result. Chi square analysis (Χ^2^ = 30.06) of the positives and false positives generated by both sample populations indicated a significant difference at the 95% confidence level.

## 4. Discussion

Listeria environmental screening continues to represent a significant proportion of global *Listeria* testing carried out in the food microbiology laboratory [10]. Accordingly, facile methods to identify presumptively positive environmental samples reduce the cost and time required. Paradigm Diagnostics developed an environmental screening test to identify presumptive positive *Listeria* samples. The method has been shown to be more sensitive than the USDA method [11] and potentially avoids the risk of false negative samples due to the presence of acriflavin in the enrichment medium used by most commercial enrichment media [12].

The data set in Table 1 represent environmental samples from diverse sources of food-processing facilities, Appendix A. The data translate to a sensitivity and specificity for LIB (original formulation) of 97.2% and 86.2%, respectively. In contrast, the sensitivity and specificity data for LIB v-2.0 are 100% and 100%, respectively. The positive predictive values of the respective media are 63% for LIB and 100% for LIB v-2.0.

The field data underscore the substantially better diagnostic performance characteristics of LIB v-2.0 compared with the original LIB formulation. Furthermore, the sensitivity of the new medium appears to be comparable to or better than the original formulation. We had anticipated that the new formulation would exhibit more false negatives since LIB v-2.0 contains higher levels of lithium chloride than LIB. However, we found that the LIB v-2.0 medium exhibited a greater sensitivity, with a value of 100% versus 97.2% for LIB.

This may make sense when one considers that the growth of competitive microflora, particularly *Enterococcus* sp., may inhibit the growth of *Listeria* sp. in the sample. In a recent publication, Hanachi et al. detail the potential to use *Enterococcus* sp., especially *E. faecalis* and *E. faecium*, to control the growth of *Listeria monocytogenes* in food products [13]. In addition to *Enterococcus* sp., many species within the lactic acid bacteria family are capable of producing anti-listerial compounds. The ability of these organisms to compete with *Listeria* sp. resides in their capability to both grow more robustly and produce anti-listerial bacteriocins [14].

Appendix A provides detailed site information from which the samples were obtained at their respective facilities.

In conclusion, we have demonstrated that the new formulation of the environmental *Listeria* screening test, LIB v-2.0, exceeds the performance characteristics of the original formulation, LIB, in comparison field trials. LIB v-2.0 provides a greater accuracy and a higher positive predictive value without sacrificing the test sensitivity.

## Figures and Tables

**Table 1:** Field Trial Summary.

Medium	Total Samples	Presumptive Positives	Negatives	TP*	TN	FP	FN	Χ^2^
LIB	161	54	106	34	106	20	1	
LIBv-2.0	161	35	126	35	126	0	0	30.06

TP = true positive, TN = true negative, FP = false positive, FN = false negative. *Confirmed using MOX plating and qPCR as described in the US Food and Drug Administration Bacteriological Assay Manual [8].

## Appendix A

**Table A1:**

**Ready to Eat Food Facility**			
**Location**	**LIB**	**LIB v.2.0**	**MOX, PCR**
Cooler 1: Aisle A: Pepper Pallet	NEG	NEG	
Squeegee in Cooler 2	NEG	NEG	
Curtain between coolers 1 & 2; aisle A	NEG	NEG	
Curtain between coolers 2 & 3; aisle A	NEG	NEG	
Wood under Plate Cooler 2	NEG	NEG	
**Dampness behind Wood on floor**	**POS**	**NEG**	**NEG**
Blue CHEP pallet Cooler 3 (damp)	NEG	NEG	
Cooler 3 drain	NEG	NEG	
Wood Pallet (damp) Cooler 3	NEG	NEG	
Floor Under Rack (105) wet - cooler 3	NEG	NEG	
ICE from case of Brussel Sprouts Rack 105 Cooler 3	NEG	NEG	
Underneath Table 26; School Cooler	NEG	NEG	
**Inside of Floor Scrubber lid**	**POS**	**NEG**	**NEG**
Blue Filter of Floor Scrubber reservoir	NEG	NEG	
**Inside of Floor Scrubber hose**	**POS**	**NEG**	**NEG**
Floor Scrubber Brush	NEG	NEG	
**Dishwasher Floor Drain (Bin cleaning area)**	**POS**	**NEG**	**NEG**
**Meat Processing—Fermentation/Drying**			
**Location**	**LIB**	**LIB v.2.0**	**MOX, PCR**
Drain in packaging room	NEG	NEG	
Vacuum Machine	NEG	NEG	
Under Packaging Room table	NEG	NEG	
**Dishwater room drain**	**POS**	**NEG**	**NEG**
Underneath foot stool	NEG	NEG	
Hand sink drain	NEG	NEG	
Squeegee	NEG	NEG	
RTE room drain by ECA device	NEG	NEG	
Drain in cooked cooler	NEG	NEG	
Coving in cooked cooler	NEG	NEG	
**Smoke cart wheels**	**POS**	**NEG**	**NEG**
Black cart wheels	NEG	NEG	
Dish sink drain right	NEG	NEG	
Dish sink drain middle	NEG	NEG	
Dish sink drain left	**POS**	**NEG**	**NEG**
RTE floor drain outside aging cooler	NEG	NEG	
Raw Door Floor	**POS**	**NEG**	**NEG**
**RTE Food Facility/Sandwiches/Salads**	**LIB**	**LIB v.2.0**	**MOX, PCR**
Cooling Unit # 1	NEG	NEG	
Cooling Unit #2	NEG	NEG	
Cooling Unit #3	NEG	NEG	
Cooling Unit #5	NEG	NEG	
Cooling Unit #6	NEG	NEG	
Drain #14	NEG	NEG	
**Drain #15**	**POS**	**POS**	**Lm**
Line #3 Bag hole	NEG	NEG	
Threshold Swing Door #3	NEG	NEG	
Threshold Swing Door #2	NEG	NEG	
Threshold Swing Door #1	NEG	NEG	
Above ceiling in Wash Room	NEG	NEG	
Threshold H&C cooler door fr. St	NEG	NEG	
Drain #27	NEG	NEG	
Threshold M&C cooler door fr. Rec	NEG	NEG	
Receiving Threshold	NEG	NEG	
Drain # 9	NEG	NEG	
Threshold shipping cooler Door #2	NEG	NEG	
Mat in Hallway QA office	NEG	NEG	
**Retail Store Food Areas**			
**Deli—Back Room**	**LIB**	**LIB 2.0**	**MOX, PCR**
**Drain in front of raw chicken sink, inside**	**POS**	**POS**	**Lm**
Drain in front of 3-compartment sink, inside	NEG	NEG	
Drain in back wall underneath racks	NEG	NEG	
**Inside condenser pipe in-between racks by drain #7**	**POS**	**NEG**	**NEG**
Drain underneath food prep sink	NEG	NEG	
Mop sink	NEG	NEG	
Drain behind ice machine	NEG	NEG	
Top of dishwasher	NEG	NEG	
Drain under dishwasher (no cover)	NEG	NEG	
Drain in front of Deli cooler	NEG	NEG	
**Produce Cooler**	**LIB**	**LIB v.2.0**	**MOX,PCR**
Inside access port—drain plug—Produce cooler	**POS**	**NEG**	**NEG**
Wall in Produce cooler	NEG	NEG	
Cooling unit guard inside Produce cooler	NEG	NEG	
Frame of shelf in Produce cooler (left side)	NEG	NEG	
PRE—by drain in produce cooler - water present	NEG	NEG	
PRE—water on floor of produce cooler below box	NEG	NEG	
**Outside box of produce that was dripping bottom shelf**	**POS**	**POS**	**Lm**
hole in wall right side middle	NEG	NEG	
shelf leg by floor right side	NEG	NEG	
shelf leg by door	NEG	NEG	
bottom shelf where iced produce sits	NEG	NEG	
middle shelf where iced produce sits	NEG	NEG	
shelf where organic produce sits	NEG	NEG	
coving on left side by iced produce	NEG	NEG	
hole in wall left side by iced produce	NEG	NEG	
water on floor where cut fruit sits	NEG	NEG	
**Deli (Front Room)**	**LIB**	**LIB v.2.0**	**MOX,PCR**
Food prep sink drain + underneath cover	NEG	NEG	
Drain underneath Combi Oven (cover)	NEG	NEG	
Drain under Food Prep Sink	NEG	NEG	
**Café**	**LIB**	**LIB v.2.0**	**MOX,PCR**
**Drain under soda fountain**	**POS**	**NEG**	**NEG**
Drain in front of dishwasher	NEG	NEG	
Drain under 3-compartment sink	NEG	NEG	
Drain under prep sink	NEG	NEG	
Drain by mop sink	NEG	NEG	
mop sink	NEG	NEG	
**Coffee Shop**	**LIB**	**LIB v.2.0**	**MOX,PCR**
Drain under sink	NEG	NEG	
Foam drain for coffee maker machine	NEG	NEG	
Drain under milk/coffee bar	NEG	NEG	
**Meat Plant (2)**			
**Location**	**LIB**	**LIB v.2.0**	**MOX,PCR**
Meat Rack for snack sticks	NEG	NEG	
Drain Oven Room	**POS**	**POS**	***L. mono***
Door out of oven room	**POS**	**POS**	***L. mono***
Cooler Floor	**POS**	**POS**	**L. inocua**
Packaging table	NEG	NEG	
RTE tub	NEG	NEG	
Snack Stick Cutter	**POS**	**POS**	***L. welshmeri***
**Ready to Eat Food Facility (2)**			
**Environmental Swabs—pre-op**			
**Location**	**LIB**	**LIB v.2.0**	**MOX,PCR**
Drain G cover	NEG	NEG	
Line 4 bearing on sprocket shaft	**POS**	**POS**	**L. inocua**
Line 4 good bearings	**POS**	**POS**	***L. mono***
prep room floor grate	NEG	NEG	
floor scrubber	**POS**	**POS**	***L. mono***
air hose composite	NEG	NEG	
prep room center drain	NEG	NEG	
squeegee in production	**POS**	**POS**	***L. mono***
squeegee in production	NEG	NEG	
squeegee in prep room	**POS**	**POS**	***Listeria* sp**
prep room meat and cheese carts	NEG	NEG	
Floor under racking	**POS**	**POS**	***L. mono***
Floor near prep room wall interface	**POS**	**POS**	***Listeria* sp**
Center Floor composite	NEG	NEG	
Drain composite N	NEG	NEG	
Drain composite S	**POS**	**NEG**	**negative**
Fork lift with scale	**POS**	**POS**	**L. inocua**
Fork lift (stand up)	NEG	NEG	
Cimpl Bologna Pallet	**POS**	**POS**	***Listeria* sp**
Cimpl Bologna Cardboard	NEG	NEG	
Cimpl Bologna Plastic	**POS**	**POS**	***Listeria* sp**
ASE Ham Pallet	**POS**	**NEG**	**negative**
ASE Ham Cardboard	**POS**	**NEG**	**negative**
ASE Ham Plastic	NEG	NEG	
Abbyland Pallet	**POS**	**POS**	***Listeria* sp**
Abbyland Cardboard	**POS**	**POS**	***Listeria* sp**
Abbyland Plastic	NEG	NEG	
Hot Ham pallet	**POS**	**NEG**	**negative**
Toby 409/AKA T2	**POS**	**POS**	***L. mono***
line 4 bearing (all)	**POS**	**POS**	***L. mono***
Line 3 bearing (all)	NEG	NEG	
Line 5 bearings (all)	NEG	NEG	
**Bakery**			
**Location**	**LIB**	**LIB v.2.0**	**MOX,PCR**
Dairy (“Meat“) cooler condenser pipe	**POS**	**NEG**	**negative**
Dairy (“Meat“) cooler drain	**POS**	**POS**	***L. mono***
Bakery cooler drain	**POS**	**NEG**	**negative**
Bakery cooler condenser pipe	NEG	NEG	
Drain in center of bakery room	**POS**	**POS**	***L. mono***
Drain at end of bakery cooler	**POS**	**POS**	***L. mono***
Long red drain in sandwich prep area	NEG	NEG	
Sandwich cooler condenser pipe	**POS**	**POS**	***L. mono***
Sandwich cooler drain	**POS**	**NEG**	**NEG**
Drain in middle of sandwich prep area	NEG	NEG	
“Fast chill“ condenser pipe	NEG	NEG	
“Fast chill“ drain	**POS**	**POS**	***L. mono***
Holding cooler condenser pipe	**POS**	**POS**	***L. mono***
Holding cooler drain	**POS**	**POS**	***L. mono***
Far left “finished product“ cooler condenser pipe	NEG	NEG	
Far left “finished product“ cooler drain	**POS**	**NEG**	**negative**
Far right “finished product“ cooler condenser pipe	**POS**	**NEG**	**negative**
Far right “finished product“ cooler drain	**POS**	**NEG**	**negative**
Inside tub of floor scrubber	NEG	NEG	
Inside of hose out the top of floor scrubber	NEG	NEG	
Scrub brush on bottom of floor scrubber	**POS**	**NEG**	**negative**
Scrub brush on bottom of floor scrubber	NEG	NEG	
Squeegee on back of floor scrubber	**NEG**	**POS**	***Listeria* sp**

List of abbreviations: MOX: Modified Oxford Medium, PCR: Polymerase Chain Reaction, PDX-LIB: Paradigm Diagnostics’ *Listeria* Indicator Broth. Items bold permit easier identification of positive samples in table.
